# Antimicrobial resistance genes in *Salmonella* and *Escherichia coli* isolates from chicken droppings in Nairobi, Kenya

**DOI:** 10.1186/s13104-019-4068-8

**Published:** 2019-01-14

**Authors:** Lydia Mali Langata, John M. Maingi, Harry Asena Musonye, John Kiiru, Anthony Kebira Nyamache

**Affiliations:** 1 Department of Microbiology, Kenyatta University, P.O Box 43844, Nairobi, 00100 Kenya; 2 Centre of Microbiology Research, Kenya Medical Research Institute, P.O Box 54840, Nairobi, 00200 Kenya

**Keywords:** Beta-lactamase genes, Class 1 integrons, Clonal expansion

## Abstract

**Objective:**

Increase in antimicrobial resistance is a threat to health sector globally. Surveillance on the spread and emergence of antimicrobial resistance is therefore invertible. This study investigated prevalence of *Salmonella* and *Escherichia coli*, molecularly characterized their antimicrobial resistance patterns and spread among resistant isolates from chicken droppings.

**Results:**

A total of 150 chicken households were selected randomly within Nairobi and fresh chicken droppings collected. *Salmonella* and *Escherichia coli* were isolated and antimicrobial susceptibility test carried out. Beta-lactamase genes and class 1 integrons were determined among amoxicillin resistant isolates. Isolates carrying TEM gene were further subjected to (GTG)_5_ PCR genotyping. Of the analysed samples, 57% and 12% contained *Escherichia coli* and *Salmonella* respectively. Most of the isolates were susceptible to the tested antibiotics with exemption of 53% of the isolates that were resistant to amoxicillin. The isolates were detected with TEM (46%), CTX-M (18%) resistance genes and class 1 integrons (25%). The study reveals presence of beta-lactamase genes and class 1 integrons across *Salmonella* and *Escherichia coli* isolates from droppings of reared chicken. Therefore, the wide distribution of chicken and their fecal waste is likely to increase development of antibiotic resistance.

**Electronic supplementary material:**

The online version of this article (10.1186/s13104-019-4068-8) contains supplementary material, which is available to authorized users.

## Introduction

Increase in drug resistance to frequently used antimicrobial agents in human and animal production is a public health challenge globally [1, 2]. It is estimated that by 2050, drug resistant infections are likely to cause death of 10 million people annually, if the current trends of antimicrobial resistance persist [1]. Regardless of this, global efforts to address this have generally been slow and inadequate to combat antimicrobial resistance [3]. Worldwide, there has been an increase in consumption of antibiotics [4]. Currently, the world health organization has reported high levels of antimicrobial resistance, indicating a strong correlation with the scale of antibiotic consumption [5]. The reports have indicated high antibiotic resistance levels in *Escherichia coli* and *Salmonella* among other bacteria. Nevertheless, other factors like suboptimal treatments, self-prescriptions, drug non adherences, have also promoted development of drug resistance [6].

In the recent past, antibiotics have been used widely in poultry production as growth promoters, prophylaxis and therapeutics [7]. Due to increase in demand for poultry, production of poultry could increase yearly by 3.6% in developing countries up to 2030 [8]. This suggests that the intensive poultry keeping will simultaneously increase use of antibiotics more than in human use [9]. It can also be observed that with disposal of poultry products in the environment, poor hygiene and ability of bacteria to remain in the environment, all this could likely contribute to dissemination of drug resistance variants strains [7, 10].

In Kenya, as in most of the developing countries, poultry farming is one of the widespread livestock enterprises. Survey studies have revealed that Kenya has approximately 37 million birds. It is reported that about 65% of Kenyan households keep chicken, with Nairobi having the highest number of chicken kept per household [11]. The chicken are often administered antibiotics for prevention and treatment of diseases [12, 13]. Previous studies on antimicrobial resistance have revealed high resistance levels in bacterial isolates from rectal swabs of Kenyan chicken [14, 15]. However, much less has been investigated on the associated antimicrobial resistance genes.

This study determined prevalence of *Salmonella* and *E. coli*, their antimicrobial resistance patterns, associated resistance genes, class 1 integrons and clonal expansion of resistant isolates from chicken droppings in Nairobi, Kenya.

## Main text

### Methods

#### Sampling

Between September and December 2017, 150 chicken households were randomly selected from slum settings of Kawangware, Kibera, Mukuru, Kariobangi, Dandora and Mathare in Nairobi, Kenya. From each household, fresh chicken dropping samples were collected. Based on previous prevalence reported in a related study [14], the number of samples collected was adequate to ensure isolation of *Escherichia coli* and *Salmonella.* Ethics approval was not required for there was no direct contact with the chicken. The chicken droppings were collected from the environment. The study was approved by Kenya National Commission for Science, Technology and Innovation. Informed verbal consent was obtained from farmers rearing chicken to allow samples to be collected from their households. The sampled chicken droppings were from broilers, layers and those caged within the sampled households.

#### Isolation and identification of *Salmonella* and *Escherichia coli*

Approximately 10 g of each sample of chicken droppings were enriched overnight in selenite F broth and buffered peptone broth. Selenite enriched samples were subcultured onto Xylose Lysine Deoxycholate while buffered peptone enriched samples inoculated onto Eosin Methylene Blue agar media and the cultures incubated at 37 °C for 24 h. These organisms were further confirmed with biochemical tests as earlier described [16, 17].

#### Antimicrobial susceptibility test

Disc diffusion method was used to determine the susceptibility of isolates to antibiotics [18]. The following antimicrobial agents; gentamicin (10 µg), streptomycin (10 µg), amoxicillin (30 µg), nalidixic acid (30 µg), ciprofloxacin (30 µg), chloramphenicol (30 µg), co-trimoxazole (25 µg) and tetracycline (30 µg) were tested and *Escherichia coli* ATCC 25922 used as control. Results were interpreted in accordance with Clinical and Laboratory Standards Institute guidelines [19].

#### Bacterial DNA extraction

For the organisms that were confirmed resistant to amoxicillin, the isolates were further subjected to molecular characterization for the identification of resistance genes associated with amoxicillin resistance. The bacterial DNA was extracted by use of boiling-centrifugation method as earlier described [20], with few modifications. A loop full of overnight bacterial culture was suspended in 1000 μl of sterile distilled water and then boiled for 15 min at 95 °C in a heating block. The resulting suspension was then centrifuged for 5 min at 14,000 rpm. The supernatant was used as DNA template.

### Characterization of resistance genes and class 1 integrons

Resistance genes that encode resistance to beta-lactams; TEM (temoneira), SHV (sulphydryl variable enzyme), OXA (oxacillin hydrolyzing capabilities), CTX-M (cefotaximase-Munich) and class 1 integrons were screened as previously described [21, 22]. Polymerase chain reaction amplification was confirmed by visualization with ethidium bromide staining of the gel.

#### Genotyping of isolates carrying TEM genes

Isolates carrying TEM were analysed using (GTG)_5_ genotyping method [23]. Amplicons were electrophoresed and the fingerprint banding patterns recorded. Cluster analysis was done using GelCompar II software using UPFG arithmetic mean and dice correlation.

### Results

Over 57% of the total sampled chicken droppings were found to harbor *Escherichia coli*. However, of the 150 samples, 12% were detected with *Salmonella*.

*Escherichia coli* isolates showed resistance to all antibiotics tested except to gentamicin and ciprofloxacin. Out of the 85 *Escherichia coli* isolates, the highest resistance observed was to amoxicillin 54% and the least was 2% for nalidixic acid and chloramphenicol. Intermediates were also common in all antibiotics tested within the range of 1% to 24% (Table 1).

**Table 1:** Antimicrobial susceptibility profiles of *Escherichia coli*

Antibiotics	Susceptible No. (%)	Intermediate No. (%)	Resistant No. (%)
Streptomycin	64 (75)	13 (15)	8 (9)
Gentamicin	83 (98)	2 (2)	0 (0)
Chloramphenicol	69 (81)	14 (17)	2 (2)
Nalidixic acid	71 (84)	12 (14)	2 (2)
Ciprofloxacin	84 (99)	1 (1)	0 (0)
Tetracycline	55 (65)	20 (24)	10 (12)
Amoxicillin	23 (27)	16 (19)	46 (54)
Co-trimoxazole	52 (61)	11 (13)	22 (26)

The percentage of resistance to the antibiotics differed among the *Salmonella* isolates, 50% of the isolates were resistant to amoxicillin. Resistance to co-trimoxazole, tetracycline and streptomycin was 28%, 11% and 6% respectively. None of the isolates were resistant to gentamicin, nalidixic acid, ciprofloxacin and chloramphenicol. A higher percentage of intermediate isolates 39% was observed in amoxicillin (Table 2).

**Table 2:** Antimicrobial susceptibility profiles of *Salmonella*

Antibiotics	Susceptible No. (%)	Intermediate No. (%)	Resistant No. (%)
Streptomycin	12 (67)	5 (28)	1 (6)
Gentamicin	18 (100)	0 (0)	0 (0)
Chloramphenicol	18 (100)	0 (0)	0 (0)
Nalidixic acid	17 (94)	1 (6)	0 (0)
Ciprofloxacin	18 (100)	0 (0)	0 (0)
Tetracycline	11 (61)	5 (28)	2 (11)
Amoxicillin	2 (11)	7 (39)	9 (50)
Co-trimoxazole	8 (44)	5 (28)	5 (28)

Among the bacteria screened for resistance genes, TEM and CTX-M were present. SHV and OXA were not detected among the resistant isolates. The TEM genes were detected in 46% of the 55 resistant isolates analysed. The TEM gene predominated followed by CTX-M 18% out of 55 isolates. Integrase genes in class 1 integrons were detected in 26% isolates among the 55 isolates tested (Table 3) (Additional file 1: Figure S1).

**Table 3:** Carriage of antimicrobial resistance genes (number, %) among the isolates

	Distribution of antimicrobial resistance genes among 55 isolates						
	TEM only	TEM + CTX-M	All TEM	CTX-M only	All CTX-M	OXA	SHV
Presence of class 1 integrons	10	2	12	2	4	0	0
Absence of class 1 integrons	11	2	13	4	6	0	0
Total	21 (38%)	4 (7%)	25 (46%)	6 (11%)	10 (18%)	0	0

Isolates type-able were evaluated to determine their genetic relatedness. Some isolates had different (GTG)_5_ patterns while others possessed similar (GTG)_5_ patterns. Construction of dendogram from (GTG)_5_ profiles of the isolates, separated the isolates into various clusters. Some of the isolates were clustered together despite from different sampling sites (Additional file 2: Figure S2 and Additional file 3: Figure S3).

### Discussion

The results showed 57% prevalence of *E. coli* in chicken droppings obtained from broilers and layers. This differed from other related studies. In Kenya, a prevalence of 67% and 100% was reported in rectal swabs of indigenous and caecal samples of broiler chicken respectively [14, 24]. Prevalence of 99% was reported in Grenada [25]. Mixed infection of samples with other microbes has been reported to affect prevalence of some bacteria such as *E. coli* and this could possibly be the cause of different prevalence from other studies. Environmental conditions, geographical location and other host factors affect prevalence of *E. coli* among related animals and could also account for the differences [26].

The study revealed 12% occurrences of *Salmonella* in collected samples. The detection level of *Salmonella* was higher compared to related study carried out in Kenya. The study reported a prevalence of 3.6% in chicken rectal swabs [15]. The variation in prevalence can be related to difference in hygiene practices among the chicken farmers. In addition, direct transmission of the bacteria from humans as well as difference in contamination levels of poultry feeds may be used to justify. In contrast to this study, other studies have reported prevalence higher than 20%. In Uganda and Nigeria, prevalence of 21% and 23% were observed respectively [27, 28]. Studies carried out in Burkina Faso and Gambia reported prevalence of 55% and 67% respectively [29, 30]. The prevalence of *Salmonella* in poultry has been demonstrated to differ depending on country, the type of production methods and measures used to control contamination [29].

Different resistance patterns of *Escherichia coli* and *Salmonella* isolates to antibiotics tested in the current study are in consistence with previous related studies on antimicrobial resistance [31–33]. The patterns reflect resistance to commonly used antibiotics including amoxicillin, co-trimoxazole, tetracycline, streptomycin and low resistance to ciprofloxacin and chloramphenicol. In Kenya, studies have reported extensive use of tetracycline and sulphonamides such as co-trimoxazole in poultry [12, 13]. Despite this, similar to the current study, studies have reported resistance to beta-lactams in addition to tetracycline and co-trimoxazole [14, 15, 25]. Moreover, the current study recorded higher resistance to amoxicillin. This demonstrates that apart from therapeutic use of antibiotics in poultry, other factors influence development of antibiotic resistance.

Beta-lactamase genes were detected among the isolates. These genes have previously been detected in poultry [34–36]. The TEM gene predominated followed by CTX-M; this was similar to previous study in Nigeria that reported prevalence of 63% and 35% of TEM and CTX-M respectively in isolates from chicken droppings [34]. In contrast, high prevalence of OXA has been reported in Algeria while in Germany the predominant beta-lactamase gene was CMY-2 and SHV [37, 38]. In Zambia, the most prevalent was CTX-M. This justify that resistance gene prevalence in bacterial isolates from poultry varies across the world [36]. Even though resistance to amoxicillin is significantly related to the presence of a beta-lactamase gene, some isolates that were resistant to this antibiotic did not have the resistance genes. This could imply that some of the isolates carry beta-lactamase genes different from the ones that were screened in this study. Since most of the common beta-lactamase genes were screened, resistance to amoxicillin could have been caused by other resistance mechanisms. The detected beta-lactamase genes, among reared chicken for food, pose a threat to disease infections caused by these organisms and related. These findings suggest that the high rate of disposal of chicken droppings in the environment is likely to accelerate the rate of spread of antimicrobial resistance genes.

Most of the class 1 integrons were detected in beta-lactamase positive isolates. The results of this study support previous findings that some of beta-lactamase genes are located within integron as gene cassettes [21, 39, 40]. The results demonstrate that integrons are widespread among bacterial communities in chicken droppings. The genetic context of class 1 integron is linked with dissemination of antibiotic resistance genes [41]. Therefore, presence of these integrons in the current study suggests high rate of transfer of resistance genes from chicken droppings.

Similarities in genetic profiles among *E. coli* and *Salmonella* isolates carrying TEM gene from different locations may be linked to clonal expansion of these bacteria. Some resistant genes have been reported to spread through clonal expansion of antimicrobial resistant bacteria [42, 43]. However, some of the bacterial isolates were not closely related when subjected to (GTG)_5_ PCR typing despite from the same sampling site. This implies that the resistance genes could have been gained through independent genetic mechanisms, possibly selection for resistance following exposure to antibiotics and through horizontal gene transfer. Previously, resistance caused by expansion of clones and independent strains has been described [42]. This indicated that antimicrobial resistance is a consequence of complex interactions involved in spread of resistance.

## Limitation

The current study never determined the specific source of the resistance genes. It is important that further investigation be carried out to establish the source. Their acquisition could be through cross contamination from humans, prior exposure to antibiotics or from contaminated poultry feeds by resistant microorganisms.

## Additional files

**Additional file 1. Figure S1.** PCR detection of TEM, CTX-M and integrase gene in class 1 integrons.

**Additional file 2. Figure S2.** Dendogram of TEM positive *Escherichia coli* isolates.

**Additional file 3. Figure S3.** Dendogram of TEM positive *Salmonella* isolates.

## Abbreviations

TEM: temoneira
OXA: oxacillin hydrolysing capabilities
SHV: sulphydryl variable enzyme
CTX-M: cefotaximase-Munich hydrolysing capabilities
