# Supplementary material for: Antimicrobial resistance genes in Salmonella and Escherichia coli isolates from chicken droppings in Nairobi, Kenya
Source: BMC Res Notes. 2019 Jan 14;12:22. doi: 10.1186/s13104-019-4068-8 (PMC6332563; doi:10.1186/s13104-019-4068-8)
Supplement: Supplementary file 1 — Additional file 1. Figure S1. PCR detection of TEM, CTX-M and integrase gene in class 1 integrons. [file 13104_2019_4068_MOESM1_ESM.docx]

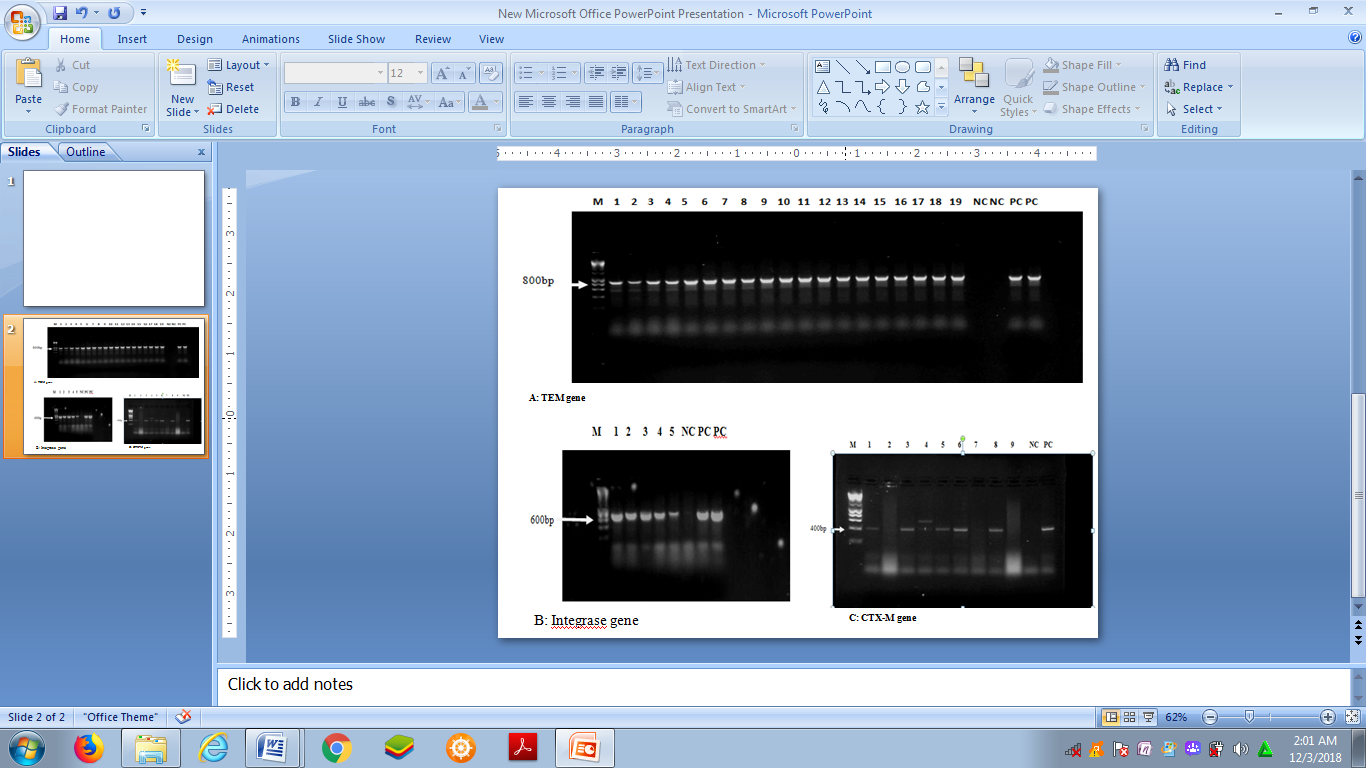


**Key**=M: 1 Kb DNA ladder, 1-19 isolates from chicken droppings, NC: Negative controls PC: Positive controls, A: TEM, B: Integrase, C: CTX-M
