# Supplementary material for: Antimicrobial resistance genes in Salmonella and Escherichia coli isolates from chicken droppings in Nairobi, Kenya
Source: BMC Res Notes. 2019 Jan 14;12:22. doi: 10.1186/s13104-019-4068-8 (PMC6332563; doi:10.1186/s13104-019-4068-8)
Supplement: Supplementary file 2 — Additional file 2. Figure S2. Dendogram of TEM positive Escherichia coli isolates. [file 13104_2019_4068_MOESM2_ESM.docx]

**
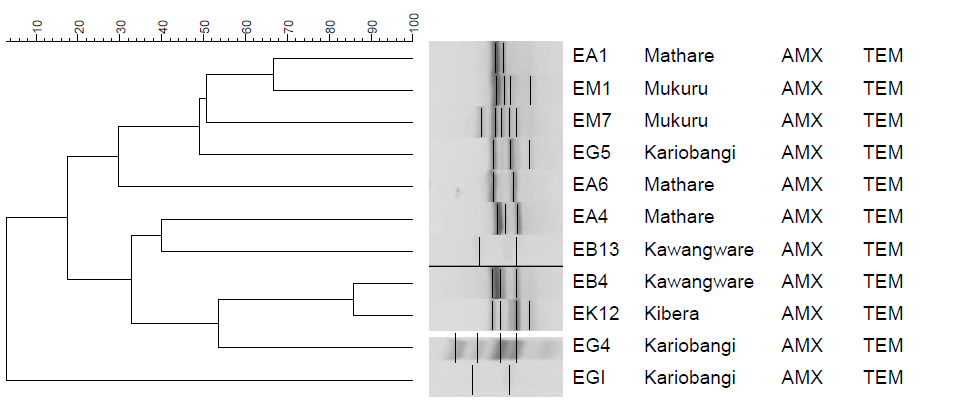
**

**Key:** (EA1, EM1, EM7, EG5, EA6, EA4, EB13, EB4, EK12, EG4, EG1) = *Escherichia coli* isolates from chicken droppings with their respective locations, AMX=amoxicillin resistance, TEM=beta-lactamase gene
