# Supplementary material for: Antimicrobial resistance genes in Salmonella and Escherichia coli isolates from chicken droppings in Nairobi, Kenya
Source: BMC Res Notes. 2019 Jan 14;12:22. doi: 10.1186/s13104-019-4068-8 (PMC6332563; doi:10.1186/s13104-019-4068-8)
Supplement: Supplementary file 3 — Additional file 3. Figure S3. Dendogram of TEM positive Salmonella isolates. [file 13104_2019_4068_MOESM3_ESM.docx]

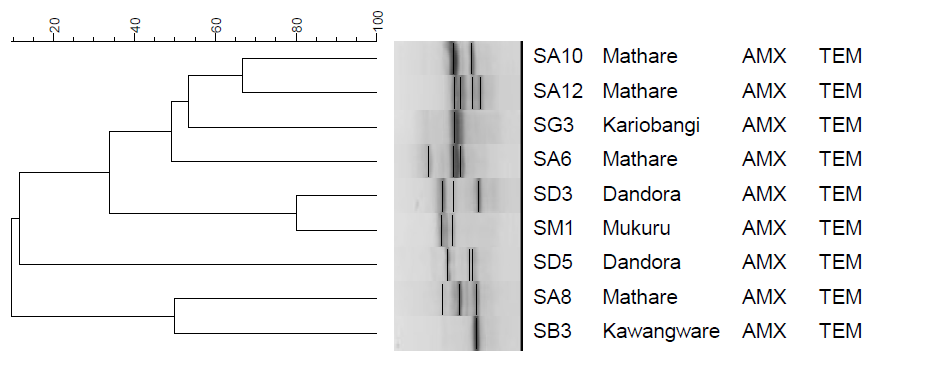


**Key:** (SA10, SA12, SG3, SA6, SD3, SM1, SD5, SA8, SB3) = *Salmonella* isolates from chicken droppings with their respective locations, AMX=amoxicillin resistance, TEM=beta-lactamase gene
